# Distance learning on breastfeeding for residents in pediatrics

**DOI:** 10.1007/s00431-025-06468-z

**Published:** 2025-09-26

**Authors:** Riccardo Davanzo, Guglielmo Salvatori, Maria Lorella Gianni, Antonio Corsello, Mariella Baldassarre, Elena Scarpato, Massimo Agosti

**Affiliations:** 1 University of Insubria, Varese, Italy; 2 Neonatal Intensive Care Unit – “Bambino Gesù” Children’s Hospital IRCCS, Rome, Italy; 3 Dipartimento Di Scienze Cliniche E Di Comunità, Università Degli Studi Di Milano, Dipartimento Di Eccellenza, 2023-2027 Milan, Italy; 4 NICU, Fondazione IRCCS Cà Granda Ospedale Maggiore Policlinico, Milan, Italy; 5 Department of Clinical-Surgical, Diagnostic and Pediatric Sciences, University of Pavia, Pavia, Italy; 6 Department of Interdisciplinary Medicine, Neonatology and NICU, Aldo Moro University, Bari, Italy; 7 Department of Translational Medical Sciences, Section of Pediatrics, University Federico II of Naples, Naples, Italy

**Keywords:** Breastfeeding, Human milk, Distance learning, Pediatric residency, Medical education

## Abstract

Breastfeeding is essential for maternal and neonatal health, yet its promotion and support are often insufficiently addressed in medical and pediatric residency curricula. This study evaluated the acceptance and perceived effectiveness of a distance learning course on breastfeeding (DLC-Bf) developed by the Italian Societies of Pediatrics and Neonatology. A cross-sectional, observational study was conducted between August and November 2024. All pediatric residents enrolled in Italian pediatric schools (*n* = 1281) were invited to participate in an 11.5-h asynchronous distance learning course. The course included pre-recorded lectures, reading materials, and clinical case discussions. A 33-question anonymous online questionnaire was administered to evaluate participants’ perceptions of the course’s quality, content, format, and usefulness. Descriptive statistics were used for data analysis. Of the 1281 residents invited, 74.2% accessed the course and 40.4% completed at least 90% of it. Among those who completed the DLC-Bf, perceived clarity, clinical relevance, and usefulness were high, particularly for modules on the management of common breastfeeding issues; however, only 23% completed the full course and 13.2% of all invitees responded to the survey, limiting inference due to potential selection and nonresponse bias. Almost two out of three residents noted discrepancies between the course contents and current practices in training hospitals, mainly referred to the management of the most common breastfeeding problems. The DLC-Bf was well received, highlighting the need for structured breastfeeding education during pediatric training. It may require improvements in order to increase the participation rate of residents, although this might depend mainly on the specific priority given to breastfeeding. Future research should assess the course’s impact on residents’ knowledge, attitudes, and skills in breastfeeding support.

**Table:** 

**What is Known:** *• Breastfeeding is fundamental for maternal and neonatal health, even if training in medical and pediatric residency programs is often inconsistent.* *• Pediatricians play a key role in supporting breastfeeding, but curricula lack standardized, evidence‑based breastfeeding education.*	
**What is New:** *• We developed an online distance learning course on breastfeeding for pediatric residents with high reported satisfaction but low full completion* *• Practice–content discrepancies were observed, indicating the need for more interactive, clinically focused modules*

**Supplementary Information:**

The online version contains supplementary material available at 10.1007/s00431-025-06468-z.

## Background

Breastfeeding is universally recognized for its health, social, and economic benefits, reducing maternal and neonatal morbidity and supporting sustainable feeding practices [1, 2]. In particular, pediatricians and neonatologists play important roles in guiding and advising mothers on infant feeding, thus possibly influencing the beginning and success of breastfeeding. However, the topic of breastfeeding is often not adequately addressed in the *curricula* of medical students and pediatric residents, and breastfeeding skills are often patchy and inconsistent [3–10]. Furthermore, these deficiencies extend beyond complex clinical situations—such as guiding preterm infants from tube feeding to direct breastfeeding—to essential breastfeeding fundamentals like latch assessment, which is crucial for supporting early breastfeeding [10, 11].

To address this gap, the Task Force on Breastfeeding of the Italian Ministry of Health, in collaboration with leading scientific societies involved in perinatal and pediatric care and the National College of Schools of Pediatrics, has developed and disseminated recommendations on breastfeeding training for future pediatricians [12]. These recommendations aim to incorporate breastfeeding education into the specific curricula of healthcare professionals [13]. Consequently, the Italian Societies of Neonatology (SIN) and Pediatrics (SIP) have developed a distance learning course on breastfeeding (DLC-Bf) tailored for pediatric residents. This cross-sectional study aimed at evaluating the perceived quality and overall acceptance of the DLC-Bf among Italian pediatric residents, providing valuable insights into its impact on their training. Beyond Italy, a recent systematic review documented persistent gaps in breastfeeding skills training across countries and professions, underscoring the need for standardized, clinically oriented education for pediatric trainees and allied staff [14].

## Materials and methods

As part of the Project for the Promotion of Breastfeeding in Italian Maternity Hospitals [15], an 11.5-h Distance Learning Course on Breastfeeding (DLC-Bf) was developed collaboratively by the Breastfeeding Sections of SIN and SIP, taking inspiration from the already available course of the United Nations Children’s Fund (UNICEF) and the Lactation Education Accreditation and Approval Review Committee (LEAARC) [16–18]. The DLC-Bf consisted of 14 modules prepared and conducted by 16 expert trainers. The course was made freely available from November 2023 to November 2024 to the 37 Italian university schools of pediatrics through a university-specific access code, inviting all the pediatric residents.

Given its nature, the practical learning component was excluded from the DLC-Bf and was entrusted to each school of pediatrics, which independently identified the most suitable healthcare facilities for internship placements. A total of 1281 pediatric residents voluntarily enrolled in the study. The survey was accessible over a four-month period, from August to November 2024.

The DLC-Bf delivered evidence-based, up-to-date content, covering topics such as the biology of human milk, the physiology of lactation, and the management of common breastfeeding problems encountered in pediatric practice (Supplementary Material 1) [13]. Trainers were selected on the basis of their expertise in lactation and breastfeeding ensuring a good standard of competence, even if adult learning techniques grounded in the principle of andragogy were very limitedly applied [19, 20]. Reading materials were provided on request.

At the end of the period of accessibility to the DLC-Bf, all registered trainees were invited by e-mail to fill out an anonymous 33-question survey hosted on SurveyMonkey (Supplementary Material 2) to obtain their opinions on the contents of the course, their usefulness and comprehensiveness, the additional need for bibliographic materials, the eventual discrepancy between the content of the course and their current clinical experience and, lastly, the appropriateness of the length of the DLC-Bf.

The survey, not previously validated, followed the Checklist for Reporting Results of Internet e-Surveys (CHERRIES), when applicable (Supplementary Material 3) [21]. The questionnaire was built up according to a 3-point or 4-point Likert scale. No sociodemographic data regarding the residents was requested and no incentives were provided to participants. No previous field test of the survey or formal validation of the e-questionnaire has been done. An informed consent sheet for the online survey clearly explained to residents the study’s purpose. The number of students who completed the course was directly obtained from the learning platform.

This cross-sectional observational study was performed in line with the principles of the Declaration of Helsinki. The study has been approved by the ethical board of SIN (Com.A. SIN, 5/12/2023). Data from the questionnaires were processed and analyzed using SPSS v27. Descriptive statistics (frequencies, percentages, means) were calculated.

## Results

Of 1281 registered residents, 950 (74.2%) accessed the course, 518 (40.4%) attended ≥ 90% of the content, and 295 (23%) completed all the modules on the learning platform (Supplementary Material 4). Of all invitees, 169 submitted the survey (13.2%), representing 57.3% of course completers. Item-level missingness occurred as some respondents skipped individual optional questions.

Overall feedback was positive (Table 1). Nearly all respondents (> 95%) rated “prevention of common breastfeeding problems” and “management of common problems in hospital/outpatient/home” as useful. Clinical case discussions were likewise highly valued. Findings show that residents valued clinically practical items (e.g., problem solving and cases) over more academic content. When asked about alignment between the course and actual practice, 60% of respondents noted some discrepancies. Only 38% felt the course entirely matched their hospital’s routines; the remaining 62% reported partial (60%) or major (2%) mismatches. In particular, 76.2% of surveyed residents cited inconsistencies in how common breastfeeding issues are generally managed in their training hospitals versus in the course recommendations.

**Table 1:** Perceived usefulness of the course topics

Contents of the online course	Useful topic	Less useful topic, but to be maintained	To be revised
• Composition and properties of human/breast milk	87.3%	10.3%	2.4%
• Anatomy of the breast and physiology of lactation	70.3%	24.9%	4.8%
• Epidemiology of breastfeeding	61.8%	26.7%	11.5%
• Personal and socioeconomic determinants of breastfeeding	64.4%	26.7%	7.9%
• Prevention of the most common problems with breastfeeding	95.8%	3.6%	0.6%
• Management (in hospital, outpatient clinic and at home) of the most common problems with breastfeeding	98.8%	1.2%	0%
• Clinical cases	93.3%	4.8%	1.8%

Few discrepancies were noted for basic topics like breast milk composition or physiology (reported by only 1–3%). Finally, over half of respondents expressed interest in enhanced interactivity and resources. More than 50% requested greater synchronous or asynchronous interaction with instructors (e.g., Q&A sessions) and improved access to bibliographic materials for in-depth study. Regarding course length, a strong majority (79.1%) found the 11.5 h duration appropriate, but 15.4% suggested shortening it (ideally to about 8 h).

**Fig. 1:**
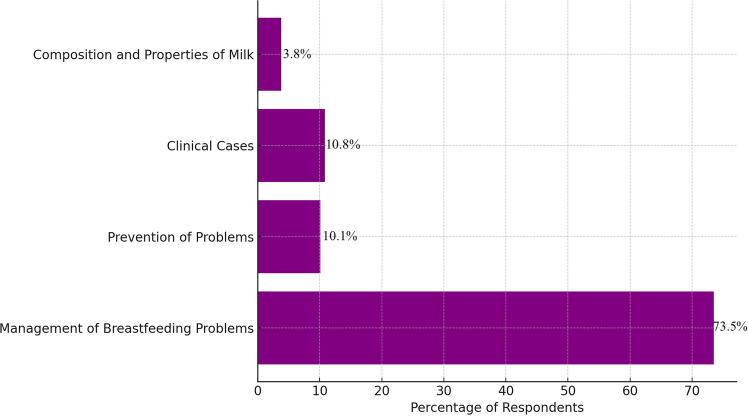
Interest for in-depth teaching on selected topics

A substantial proportion of respondents expressed a desire for further training in breastfeeding. Moreover, 73.5% of residents would like more in-depth teaching materials on the management of problems and common diseases related to breastfeeding (Fig. 1). The topics that raised the greatest interest included the management of low milk supply, difficult latching and infant crying, and breastfeeding in special situations (e.g., preterm infants, maternal infections).

## Discussion

The training gaps observed in our study are consistent with international evidence indicating insufficient, heterogeneous breastfeeding education for healthcare professionals, including pediatric trainees, which strengthens the general rationale for structured, skills-oriented curricula [14]. Inadequate training on breastfeeding of health professionals might represent one determinant of suboptimal breastfeeding rates in the population. This is particularly true for pediatricians and residents, who play a key role in informing and supporting mothers. In fact, they need in-depth training on breastfeeding not only to provide competent and appropriate advice to mothers and families but also to implement hospital and outpatient practices that are known to facilitate the mother‒child relationship and breastfeeding [22–25].

Training gaps in breastfeeding have multiple causes [14]. First, breastfeeding training programs are heterogeneous or even not present in the *curricula* of some Italian schools of pediatrics. Trainees do not always have the opportunity to observe and participate in the active management of breastfeeding-related problems because of the limited time spent in the newborn care sector. Second, the hospitals where residents are trained do not always represent a valid model of protection, promotion and support for breastfeeding [26], since many of these hospitals not have reached the UNICEF gold standard of a Baby Friendly Hospital [27]. Third, teaching staff is sometimes more competent in the biological and nutritional aspects of breastfeeding than in the effective management of breastfeeding problems. Finally, teaching materials on specific topics may not be properly selected, updated and thus didactically valid.

To cover medical educational needs related to breastfeeding while integrating theory and practice, teaching methodologies may be diverse. Thus, in addition to “in person” training, distance learning lessons, workshops, simulations and interactive clinical cases may be used [19, 20]. In particular, clinical simulation may profitably test trainees on counseling and management techniques for common breastfeeding problems in a safe and supervised setting. Finally, internship periods can be organized in hospitals dedicated to breastfeeding, such as Baby-Friendly Hospitals, or hospitals with implemented organizational models that effectively facilitate breastfeeding, such as, in Italy, the health facilities that joined the Hospital Policy on Breastfeeding Project [15].

The present study, which was conducted on a sample of Italian residents, shows their appreciation for a DLC-Bf specifically prepared by SIN and SIP. Understandably, residents were most interested in practical problems with breastfeeding, as trainees usually favor the acquisition of knowledge that allows them to learn or improve the practical management of the patient. However, residents suggest a reduction in the duration of the online course. If basic theoretical topics related to breastfeeding are routinely addressed during medical studies, guaranteeing good basic knowledge of the biology of human milk and the physiology of breastfeeding, this suggestion could be easily fulfilled.

The results of the study must be interpreted in light of several limitations. First, it must be noted that only 23% of the initially enrolled trainees completed the online course. It is not known whether this limited involvement depends on suboptimal methods of the teaching proposal (e.g., excessive duration of the online course or difficulty encountered by teachers to maintain the interest of the trainees) or whether it is a priori influenced by an inadequate value of breastfeeding in the academic environment.

Second, the participation to the DLC-Bf was voluntary, as it depends on the intention of every single University to insert this educational module into the Pediatrics curriculum and on every single school to facilitate to a variable extent the participation of residents. Italian Universities have rarely developed educational comprehensive modules on breastfeeding. Noticeably, a course such as DLC-Bf centrally prepared even by accredited Scientific Societies has difficulty to be recognized by the Universities and be promoted and incorporated into the curriculum of the local school.

Among the trainees who completed the online course, only slightly more than half provided feedback on the educational quality of the course. Responders are presumably those trainees most interested in the topic of breastfeeding, so the appreciation expressed for the online course could mainly depend on motivational selection. This study has a major limitation in participation and response: only 23% completed the course and 13.2% of invitees responded to the survey, raising substantial risks of selection and nonresponse bias. Importantly, selection likely affected both the subset who completed the course and those who answered the survey, limiting external validity and potentially inflating perceived usefulness among more motivated trainees. Voluntary participation at both the course and survey levels introduces risks of selection and nonresponse bias; no sampling frame beyond the invited cohort was used, and no weighing or statistical correction was applied given the descriptive scope.

A mean to possibly increase in the near future the participation rate of residents is to improve the DLC-Bf, introducing the following changes: (1) shortening by a couple of hours; (2) balancing the length of different lessons according to the preference expressed by residents, giving priority to the prevention and management of the most common problems with breastfeeding and to clinical cases: (3) adopting selected adult learning techniques, although there is still some controversy on the applicability and effectiveness of the “andragogic” doctrine in general education settings, and particularly in the training of medical professionals [19, 20, 28]. Indeed, the psychological motivation for medical student and resident learning is believed to be context-dependent and significantly influenced more by internal than external factors [29].

In this invited national cohort, a web-delivered structured breastfeeding course was well received by pediatric residents but had low full completion and survey response rates. Future improvements of the online course should focus on clinically oriented modules and interactivity. Moreover, the educational effectiveness of the course should also be addressed, exploring knowledge, skills, and clinical behaviors of participants.

## Supplementary Information

Below is the link to the electronic supplementary material.

Supplementary File 1 (189 KB)

## Data Availability

Data are available from the corresponding author upon reasonable request.

## References

[1] Rollins NC, Bhandari N, Hajeebhoy N et al (2016) Why invest, and what it will take to improve breastfeeding practices? Lancet 387:491–504. 10.1016/S0140-6736(15)01044-2 26869576 10.1016/S0140-6736(15)01044-2

[2] Victora CG, Bahl R, Barros AJD et al (2016) Breastfeeding in the 21st century: epidemiology, mechanisms, and lifelong effect. Lancet 387:475–490. 10.1016/S0140-6736(15)01024-7 26869575 10.1016/S0140-6736(15)01024-7

[3] Navarro I, Soriano JM, Laredo S (2021) Applying systematic review search methods to the grey literature: a review of education and training courses on breastfeeding support for health professionals. Int Breastfeed J 16:31. 10.1186/s13006-021-00373-5 33823895 10.1186/s13006-021-00373-5 PMC8025331

[4] Bahawi YO, Al-Wassia HK, Bahaidarah SA et al (2023) Are pediatric residents in Saudi Arabia equipped to provide breastfeeding care? A cross-sectional study. Saudi J Med Med Sci 11:319–325. 10.4103/sjmms.sjmms_208_22 37970454 10.4103/sjmms.sjmms_208_22 PMC10634463

[5] Esselmont E, Moreau K, Aglipay M, Pound CM (2018) Residents’ breastfeeding knowledge, comfort, practices, and perceptions: results of the breastfeeding resident education study (BRESt). BMC Pediatr 18:170. 10.1186/s12887-018-1150-7 29788928 10.1186/s12887-018-1150-7 PMC5964719

[6] Gómez Fernández-Vegue M, Menéndez Orenga M (2019) National survey on breastfeeding knowledge amongst residents in pediatrics in Spain. Rev Esp Salud Publica 93:e201908060 31368457 PMC11582919

[7] Hillenbrand KM, Larsen PG (2002) Effect of an educational intervention about breastfeeding on the knowledge, confidence, and behaviors of pediatric resident physicians. Pediatrics 110:e59. 10.1542/peds.110.5.e59 12415065 10.1542/peds.110.5.e59

[8] Albert JB, Heinrichs-Breen J, Belmonte FW (2017) Development and evaluation of a lactation rotation for a pediatric residency program. J Hum Lact 33:748–756. 10.1177/0890334416679381 28984530 10.1177/0890334416679381

[9] Biggs KV, Fidler KJ, Shenker NS, Brown H (2020) Are the doctors of the future ready to support breastfeeding? A cross-sectional study in the UK. Int Breastfeed J 15:46. 10.1186/s13006-020-00290-z 32434558 10.1186/s13006-020-00290-z PMC7238622

[10] Baerg K, Smith-Fehr J, Marko J et al (2021) Learning needs of family physicians, pediatricians, and obstetricians to support breastfeeding and inform physician education. Can Med Educ J 12:55–61. 10.36834/cmej.70049 35003431 10.36834/cmej.70049 PMC8740246

[11] Hookway L, Brown A (2023) The lactation skill gaps of multidisciplinary paediatric healthcare professionals in the United Kingdom. J Hum Nutr Diet 36:848–863. 10.1111/jhn.13172 36992632 10.1111/jhn.13172

[12] Promozione allattamento al seno. Arrivano le linee guida per la formazione degli operatori sanitari | Aogoi. https://www.aogoi.it/notiziario/archivio-news/promozione-allattamento/. Accessed 18 Jul 2025

[13] EpiCentro La formazione del personale sanitario sull’allattamento. https://www.epicentro.iss.it/allattamento/formazione-allattamento-raccomandazioni-2020. Accessed 20 Jan 2025

[14] Mulcahy H, Philpott LF, O’Driscoll M et al (2022) Breastfeeding skills training for health care professionals: a systematic review. Heliyon 8:e11747. 10.1016/j.heliyon.2022.e11747 36468118 10.1016/j.heliyon.2022.e11747 PMC9708688

[15] Davanzo R, Salvatori G, Baldassarre M et al (2024) Promotion of breastfeeding in Italian maternity hospitals: a pre-intervention study. Ital J Pediatr 50:219. 10.1186/s13052-024-01793-9 39456093 10.1186/s13052-024-01793-9 PMC11520110

[16] Infant and young child feeding: model chapter for textbooks for medical students and allied health professionals. https://www.who.int/publications/i/item/9789241597494. Accessed 17 Jul 2025 23905206

[17] Baby-friendly Hospital Initiative training course for maternity staff: customisation guide. https://www.who.int/publications/i/item/9789240008915. Accessed 17 Jul 2025

[18] Committee LEAAR, Accreditation LE, Committee AR, et al (2022) Core curriculum for interdisciplinary lactation care. Jones & Bartlett Learning

[19] Knowles MS, Holton EF, Swanson RA (2014) The adult learner: the definitive classic in adult education and human resource development. Taylor & Francis

[20] Knapke JM, Hildreth L, Molano JR et al (2024) Andragogy in practice: applying a theoretical framework to team science training in biomedical research. Br J Biomed Sci 81:12651. 10.3389/bjbs.2024.12651 38605981 10.3389/bjbs.2024.12651 PMC11008574

[21] Eysenbach G (2004) Improving the quality of web surveys: the checklist for reporting results of internet e-surveys (CHERRIES). J Med Internet Res 6:e34. 10.2196/jmir.6.3.e34 15471760 10.2196/jmir.6.3.e34 PMC1550605

[22] Società Italiana di Pediatria (2015) Allattamento al seno e uso del latte materno/umano. https://sip.it/2015/09/15/position-statement-sullallattamento-al-seno-e-uso-del-latte-maternoumano/. Accessed 20 Jan 2025

[23] Parker MG, Stellwagen LM, Noble L et al (2021) Promoting human milk and breastfeeding for the very low birth weight infant. Pediatrics 148:e2021054272. 10.1542/peds.2021-054272 34635582 10.1542/peds.2021-054272

[24] Meek JY, Noble L (2022) Technical report: breastfeeding and the use of human milk. Pediatrics 150:e2022057989. 10.1542/peds.2022-057989 35921641 10.1542/peds.2022-057989

[25] Busch DW, Wodwaski N, Webber E, Flagg JS (2025) Mastering the BASICS: essential components of a lactation curriculum for healthcare professionals. J Pediatr Health Care 39:611–622. 10.1016/j.pedhc.2025.04.001 40327025 10.1016/j.pedhc.2025.04.001

[26] Lindacher V, Altebaeumer P, Marlow N et al (2021) European standards of care for newborn health-a project protocol. Acta Paediatr 110:1433–1438. 10.1111/apa.15712 33290600 10.1111/apa.15712

[27] Implementation guidance: protecting, promoting, and supporting breastfeeding in facilities providing maternity and newborn services: the revised Baby-friendly Hospital Initiative 2018. https://www.who.int/publications/i/item/9789241513807. Accessed 20 Jan 2025

[28] Aulakh J, Wahab H, Richards C et al (2025) Self-directed learning versus traditional didactic learning in undergraduate medical education: a systemic review and meta-analysis. BMC Med Educ 25:70. 10.1186/s12909-024-06449-0 39815233 10.1186/s12909-024-06449-0 PMC11737201

[29] Misch DA (2002) Andragogy and medical education: are medical students internally motivated to learn? Adv Health Sci Educ Theory Pract 7:153–160. 10.1023/a:1015790318032 12075147 10.1023/a:1015790318032

