# Supplementary material for: Distance learning on breastfeeding for residents in pediatrics
Source: Eur J Pediatr. 2025 Sep 26;184(10):640. doi: 10.1007/s00431-025-06468-z (PMC12474732; doi:10.1007/s00431-025-06468-z)
Supplement: Supplementary file 1 — (189 KB) [file 431_2025_6468_MOESM1_ESM.docx]

**Supplementary Material 1.** *Contents of the DLC-Bf*

1. **Composition and properties of human milk**

- Human milk microbiome
- Benefits of breastfeeding for mothers, infants, families and society
- Risks of not breastfeeding for mothers and infants

1. **Anatomy of the breast, physiology of lactation, and breastfeeding**
2. **Personal and socioeconomic determinants of breastfeeding**

- Maternal physical and mental health
- Role of the partner and the family
- Breastfeeding protection, promotion and support: health policies and initiatives
- Ethical issues and laws
- Organization of health facilities: facilitating factors and obstacles
- Breastfeeding monitoring

1. **Management of common breastfeeding problems (in hospitals, clinics and at home)**

- Collecting breastfeeding history
- How to communicate with mothers
- Skin-to-skin contact and the beginning of breastfeeding
- Effective and appropriate latching onto the breast
- Ankyloglossia
- Nipple fissures, engorgement, mastitis, breast abscess, Candida
- Breast milk expression (hand expression, pumping)
- Human milk storage
- Bedsharing, co-sleeping, sudden unexpected postnatal collapse
- Breastfeeding obstacles and contraindications
- Medications and the breastfeeding mother
- Alternative devices to bottle feeding (cup, syringe, supplementary nursing system)
- Nutrition for the breastfeeding mother
- Appropriate use of formula milk
- Nipple shields

1. **Clinical cases**

- Difficult latch-on
- Low milk production
- Breastfeeding the newborn at risk of hypoglycemia
- Neonatal weight loss and infant growth
- Feeding the jaundiced newborn
- Feeding the preterm neonate
- Breastfeeding and maternal infections
- “Excessive” crying

**Supplementary Material 2.** *Questionnaire.*

## 1) The topics covered during the session were:

|  | Strongly Disagree | Slightly Disagree | Somewhat Agree | Strongly Agree |
| --- | --- | --- | --- | --- |
| Interesting |  |  |  |  |
| Clear |  |  |  |  |
| Addressed what I wanted to learn |  |  |  |  |
| Appropriate for my level of knowledge |  |  |  |  |
| Useful for my professional activity |  |  |  |  |

## 2) How useful did you find the following topics?

|  | Useful | Less useful, but should be kept in case of course revision | Less useful, but should be revised for future course editions |
| --- | --- | --- | --- |
| Composition and functional properties of breast milk |  |  |  |
| Anatomy of the breast, physiology of lactation |  |  |  |
| Breastfeeding epidemiology |  |  |  |
| Personal and socio-economic determinants of breastfeeding |  |  |  |
| Prevention (in hospital, outpatient clinic, at home) of the most common breastfeeding issues |  |  |  |
| Management (in hospital, outpatient clinic, at home) of common breastfeeding issues |  |  |  |
| Clinical cases |  |  |  |

## 3) After attending the course, did you notice any discrepancies between what you learned and what you observed colleagues doing in your professional practice?

☐ No discrepancies
☐ Partial discrepancies
☐ Significant discrepancies

## 4) If you answered B or C, which topic showed the greatest discrepancy?

|  | Number | Percentage |
| --- | --- | --- |
| Composition and functional properties of breast milk |  |  |
| Anatomy of the breast, physiology of lactation |  |  |
| Breastfeeding epidemiology |  |  |
| Personal and socio-economic determinants of breastfeeding |  |  |
| Prevention (in hospital, outpatient clinic, at home) of the most common breastfeeding issues |  |  |
| Management (in hospital, outpatient clinic, at home) of common breastfeeding issues |  |  |
| Clinical cases |  |  |

## 5) During the session you attended, would you have liked to have:

|  | Strongly Disagree | Slightly Disagree | Somewhat Agree | Strongly Agree |
| --- | --- | --- | --- | --- |
| The ability to ask questions and/or share personal experiences asynchronously (e.g., via email exchange) |  |  |  |  |
| The ability to ask questions and/or share personal experiences synchronously (live meeting with instructors) |  |  |  |  |
| More time dedicated to in-depth exploration of certain topics |  |  |  |  |
| More supplementary materials |  |  |  |  |

Other: __________________________

## 6) For which topic would you have liked to receive more supplementary materials?

|  | Number | Percentage |
| --- | --- | --- |
| Composition and functional properties of breast milk |  |  |
| Anatomy of the breast, physiology of lactation |  |  |
| Breastfeeding epidemiology |  |  |
| Personal and socio-economic determinants of breastfeeding |  |  |
| Prevention (in hospital, outpatient clinic, at home) of the most common breastfeeding issues |  |  |
| Management (in hospital, outpatient clinic, at home) of common breastfeeding issues |  |  |
| Clinical cases |  |  |

## 7) Do you think the course duration was:

☐ Too short
☐ Adequate
☐ Too long

## 8) If you answered A or C, what do you think would be the most appropriate course duration?

☐ <6 hours
☐ 6-8 hours
☐ 10-12 hours
☐ >12 hours

**Supplementary Material 3.** *CHERRIES checklist.*

| ***Checklist Item*** | ***Explanation*** | ***Page*** |
| --- | --- | --- |
| Describe survey design | Cross-sectional, observational study described in Materials and Methods. | 5 |
| IRB approval | Approved by the ethical board of SIN (Com.A. SIN, 5/12/2023) | 5 |
| Informed consent | Online sheet, voluntary participation | 5 |
| Data protection | Confidentiality and anonymity guaranteed by the platform server and the absence of personal data collection | 5 |
| Development and testing | No previous field test of the survey or formal validation of the e-questionnaire has been done. | 5 |
| Open survey versus closed survey | Restricted to pediatric residents enrolled in Italian Schools of Pediatrics | 4 |
| Contact mode | Unique link to the online survey was assigned to each university | 4 |
| Advertising the survey | Direct mailing without banner ads or use of media | 5 |
| Web/E-mail | Online survey (SurveyMonkey) | 5 |
| Context | SurveyMonkey is a website for constructing, storing and analyzing online surveys. The administrator can design the length, the kind of information provided and the type of questions & answers. The number of Italian residents restricted the maximal number of participants. The website appearance is neutral and is not suggesting any opinion, which was also true for the mailing design. | 4-5 |
| Mandatory/voluntary | Some questions not answered, indicating optional responses. | 5-6 |
| Incentives | No incentives of any kind. | 5 |
| Time/Date | The course was made freely available from November 2023 to November 2024. | 4 |
| Randomization of items or questionnaires | No randomization | 5 |
| Adaptive questioning | Not reported. | N/A |
| Number of Items | 33 questions. | 5 |
| Number of screens (pages) | 3-point or 4-point Likert scales distributed on one page. | 5 |
| Completeness check | Not applicable. The number of students who completed the course was directly obtained from the learning platform. | 5 |
| Review step | Not possible. | N/A |
| Unique site visitor | N/A | N/A |
| View rate (Ratio of unique survey visitors/unique site visitors) | N/A | N/A |
| Participation rate (Ratio of unique visitors who agreed to participate/unique first survey page visitors) | 950/1281 = 74.2%. | 5 |
| Completion rate (Ratio of users who finished the survey/users who agreed to participate) | 295/1281 = 23.0%. | 5 |
| Cookies used | Unique link with specific access code was assigned to each university. No specific cookies. | 4 |
| IP check | IP check was not conducted as unique link was used to avoid duplicate entries from the same user | N/A |
| Log file analysis | Log file analysis was not conducted as unique link was used to avoid duplicate entries from the same user | N/A |
| Registration | Unique ID was associated with a unique link. The unique ID was linked to the university information using a master log | N/A |
| Handling of incomplete questionnaires | Some participants did not answer all questions (see results) | 6 |
| Questionnaires submitted with an atypical timestamp | Not applicable. | N/A |
| Statistical correction | Descriptive statistics (see methods) | 5 |

**Supplementary Material 4.** *Flow chart on the access and participation of residents in pediatrics to the DLC-Bf.*

**
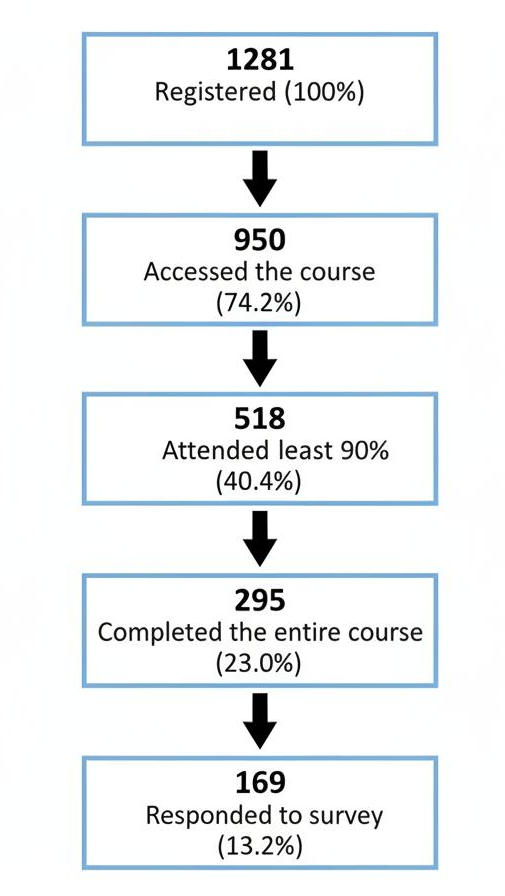
**
